# Pre-clinical pharmacology and mechanism of action of SG3199, the pyrrolobenzodiazepine (PBD) dimer warhead component of antibody-drug conjugate (ADC) payload tesirine

**DOI:** 10.1038/s41598-018-28533-4

**Published:** 2018-07-11

**Authors:** John A. Hartley, Michael J. Flynn, John P. Bingham, Simon Corbett, Halla Reinert, Arnaud Tiberghien, Luke A. Masterson, Dyeison Antonow, Lauren Adams, Sajidah Chowdhury, David G. Williams, Shenlan Mao, Jay Harper, Carin E. G. Havenith, Francesca Zammarchi, Simon Chivers, Patrick H. van Berkel, Philip W. Howard

**Affiliations:** 1 Cancer Research UK Drug DNA Interactions Research Group, UCL Cancer Institute, 72 Huntley Street, London, WC1E 6BT UK; 2 Spirogen Ltd, QMB Innovation Centre, 42 New Road, London, E1 2AX UK; 3 MedImmune, One MedImmune Way, Gaithersburg, MD 20878 USA; 4 ADC Therapeutics (UK) Limited, QMB Innovation Centre, 42 New Road, London, E1 2AX UK

## Abstract

Synthetic pyrrolobenzodiazepine (PBD) dimers, where two PBD monomers are linked through their aromatic A-ring phenolic C8-positions via a flexible propyldioxy tether, are highly efficient DNA minor groove cross-linking agents with potent cytotoxicity. PBD dimer SG3199 is the released warhead component of the antibody-drug conjugate (ADC) payload tesirine (SG3249), currently being evaluated in several ADC clinical trials. SG3199 was potently cytotoxic against a panel of human solid tumour and haematological cancer cell lines with a mean GI_50_ of 151.5 pM. Cells defective in DNA repair protein ERCC1 or homologous recombination repair showed increased sensitivity to SG3199 and the drug was only moderately susceptible to multidrug resistance mechanisms. SG3199 was highly efficient at producing DNA interstrand cross-links in naked linear plasmid DNA and dose-dependent cross-linking was observed in cells. Cross-links formed rapidly in cells and persisted over 36 hours. Following intravenous (iv) administration to rats SG3199 showed a very rapid clearance with a half life as short as 8 minutes. These combined properties of cytotoxic potency, rapid formation and persistence of DNA interstrand cross-links and very short half-life contribute to the emerging success of SG3199 as a warhead in clinical stage ADCs.

## Introduction

The pyrrolobenzodiazepines (PBDs) are a family of antitumor antibiotics which include the naturally occurring anthramycin, sibiromycin, tomaymycin, the neothramycins and DC-81^1,2^. They exert their biological activity by binding in the minor groove of DNA with a selectivity for 5′-purine-guanine-purine sequences and forming a covalent bond to the exocyclic amino group of the guanine base^3,4^.

Synthetic PBD dimers, where two PBD monomers are linked through their aromatic A-ring phenolic C8-positions via a flexible propyldioxy tether, were found to be highly cytotoxic *in vitro* and to produce DNA interstrand cross-links with high efficiency in both naked DNA and in cells^5,6^. Such molecules were shown to span six base pairs in the minor groove of DNA, covalently binding to spatially separated guanines on opposite strands in the central sequence 5′-PuGATCPy-3′ (where Pu is purine and Py is pyrimidine)^7,8^. More recent studies, using HPLC/MS and short oligonucleotides of defined sequence, have suggested that PBD dimers can also form sequence selective intrastrand cross-linked adducts and mono-alkylated adducts, in addition to interstrand cross-links^9^.

**Figure 1:**
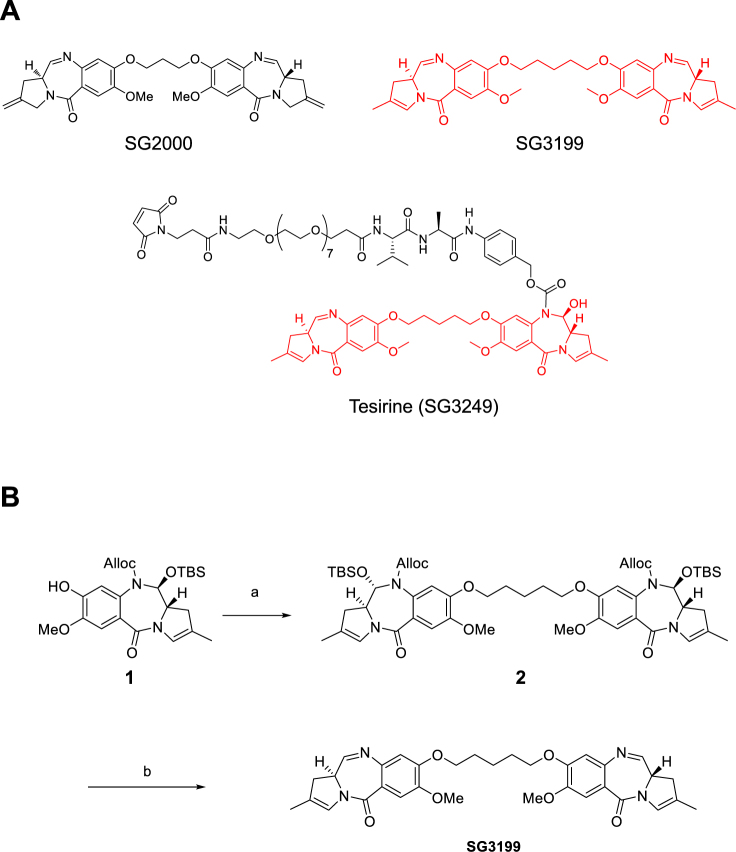
(**A**) Structures of SG2000, SG3199 and antibody-drug conjugate payload tesirine (SG3249). (**B**) Synthesis of SG3199 in two steps from phenol **1** via *bis*-alloc carbamate **2**. (a) Diiodopentane, K_2_CO_3_, Acetone, 60 °C, 18 h; (b) Pd(PPh_3_)_4_, pyrrolidine, DCM, rt, 15 min.

An important feature of the interstrand cross-links formed is that they cause minimal distortion of the DNA, a property that appears to contribute to their persistence in cells and potent biological activity^10,11^. One PBD dimer, SJG-136 (SG2000, Fig. 1A) was shown to have significant *in vitro* and *in vivo* antitumour activity^10,12^. The drug entered clinical trials against both solid tumours and haematological malignancies^13–15^.

An understanding of structure activity relationships around the PBD pharmacophore has enabled more potent PBD dimers than SG2000 to be developed. These include dimers in which the PBDs have *endo-exo* unsaturation at the C2 position (e.g. SG2202 and its prodrug SG2285^16^) and PBD dimers containing a pentyldioxy rather than a propyldioxy tether (e.g. SG2057^17^). Another example of the latter is SG3199 (Fig. 1A), a PBD dimer which is the released warhead component of the antibody-drug conjugate (ADC) payload tesirine (SG3249 Fig. 1A^18^). Rovalpituzumab tesirine (Rova-T) has completed a phase I clinical trial for the treatment of small cell lung cancer^19^ and several other ADCs incorporating payload tesirine have recently entered Phase I clinical trials including ADCT-301^20^, ADCT-402^21^, ADCT-401/MEDI3726 and ADCT-502.

The current study reports the preclinical pharmacology and mechanism of action of SG3199, confirming the properties which contribute to its success as a novel ADC warhead.

## Results

### Synthesis of SG3199

SG3199 was synthesised in two steps from monomeric phenol **1**, a building block arising during the synthesis of tesirine (Fig. 1B)^18^. Dimerisation under Williamson conditions gave bis-alloc carbamate **2** in 88% yield. Upon alloc deprotection under Deziel conditions^22^, TBS alcohol was eliminated to reveal biologically active bis-imine SG3199 in 55% yield as a white solid.

### Cytotoxicity of SG3199 against human tumour cell lines *in vitro*

**Figure 2:**
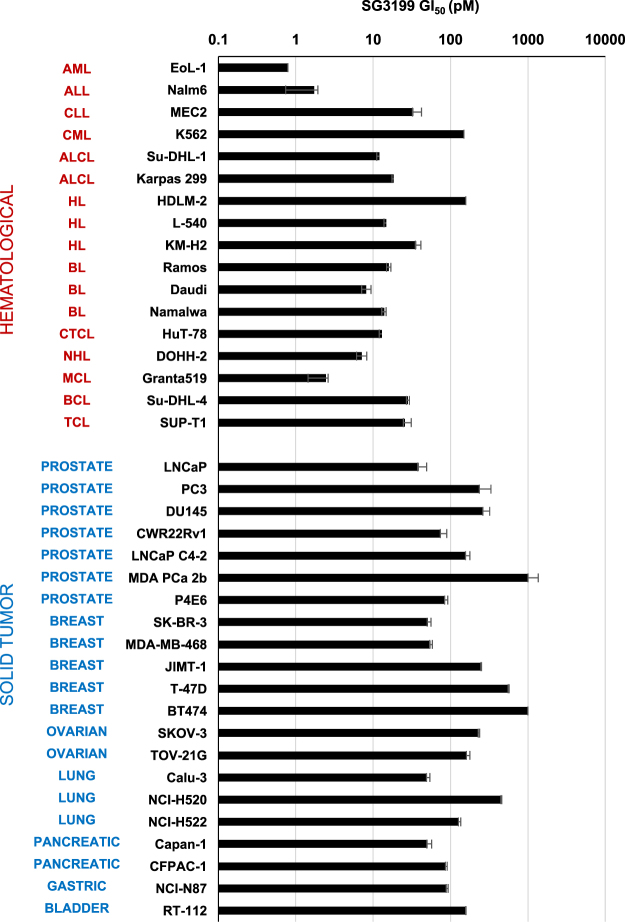
Sensitivity of a panel of human tumour haematological (red) and solid tumour (blue) cell lines to SG3199. AML-acute myeloid leukaemia, ALL – acute lymphoblastic leukaemia, CLL – chronic lymphocytic leukaemia, CML – chronic myelogenous leukaemia, ALCL – anaplastic large cell lymphoma, HL – Hodgkin lymphoma, BL- Burkitt lymphoma, CTCL – cutaneous T-cell lymphoma, NHL – non-Hodgkin lymphoma, MCL – mantle cell lymphoma, BCL – B-cell lymphoma, TCL – T-cell lymphoma.

The cytotoxicity of PBD dimer SG3199 was tested against a panel of human solid tumour and haematological cell lines. GI_50_ values, the dose of drug which inhibits cell growth by 50%, were calculated from dose response curves and are shown in Fig. 2. The GI_50_ values across the 38 cell lines ranged from 0.79 pM to 1.05 nM (mean 151.5 pM, median 74.8 pM). SG3199 was therefore potently cytotoxic in many cell lines and showed a multi-log differential activity. The 17 haematological cell lines were, in general, more sensitive to SG3199 than the 21 solid tumour cell lines with ranges of 0.79 pM to 158.6 pM (mean 31.76 pM, median 14.8 pM) and 38.7 pM to 1.05 nM (mean 248.36 pM, median 157 pM), respectively. The solid tumour cell lines represented seven organ sites and sensitivity across all sites was observed.

### Influence of DNA repair and multi-drug resistance on the *in vitro* activity of SG3199

**Figure 3:**
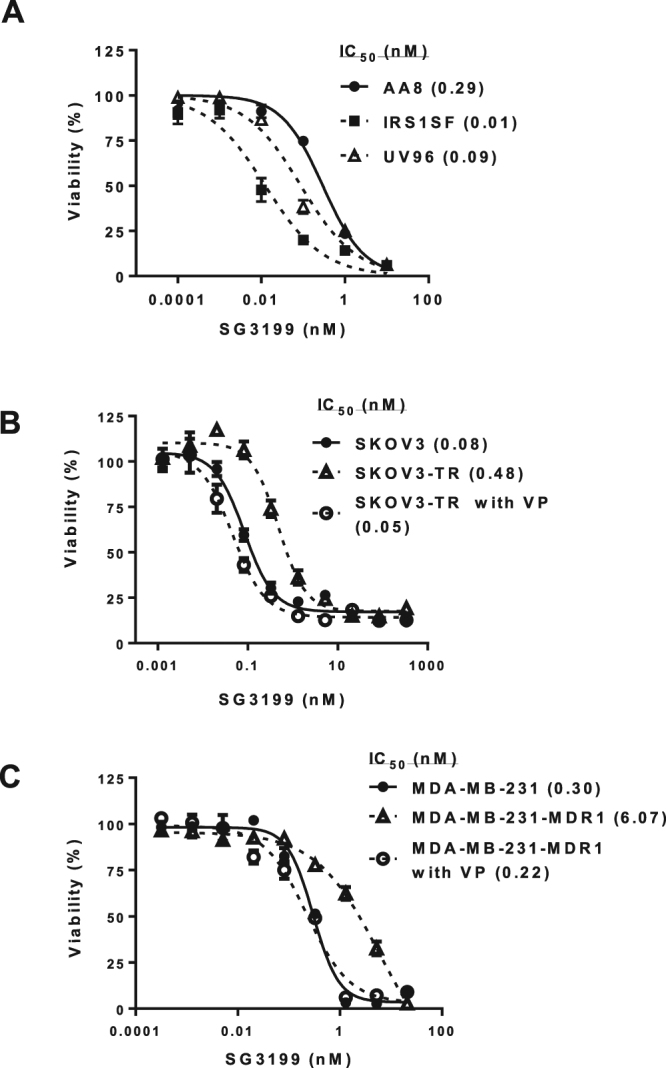
Sensitivity of SG3199 in DNA repair defective and multidrug resistant cell lines. (**A**) Chinese hamster ovarian wild type (AA8), ERCC1 defective (UV96) and homologous recombination defective (IRS1SF) cells. (**B**) Human ovarian cancer SKOV3 and SKOV3-TR cells. (**C**) Human breast cancer MDA-MB-231 and MDA-MB-231-MDR1 cells. VP is co-treatment with verapamil.

We have previously shown that defects in DNA repair protein ERCC1 and homologous recombination repair conferred sensitivity to PBD dimer SG2000^23^. The sensitivity of SG3199 in CHO cells defective in ERCC1 (UV96) and homologous recombination repair protein XRCC2 (IRS1SF) was compared to CHO wildtype AA8 cells (Fig. 3A). Increased sensitivity was observed in both DNA repair defective lines with GI_50_ values 3-fold and 30-fold for UV96 and IRS1SF cells, respectively.

Sensitivity was also assessed in paired human tumour cell lines non-expressing or expressing human multidrug resistance proteins. Chemo-resistant SKOV3-TR cells (Fig. 3B) and MDA-MB-231-MDR1 cells (Fig. 3B) were sensitive to SG3199. The difference in IC_50_ compared to the respective drug sensitive SKOV3 and MDA-MB-231 cells was 6-fold and 20-fold, respectively, indicating that SG3199 was only moderately susceptible to multidrug resistance mechanisms. A P-gp inhibitor verapamil was able to reverse the resistance in both the multidrug resistant cell lines.

### DNA interstrand cross-link formation by SG3199 in naked DNA

**Figure 4:**
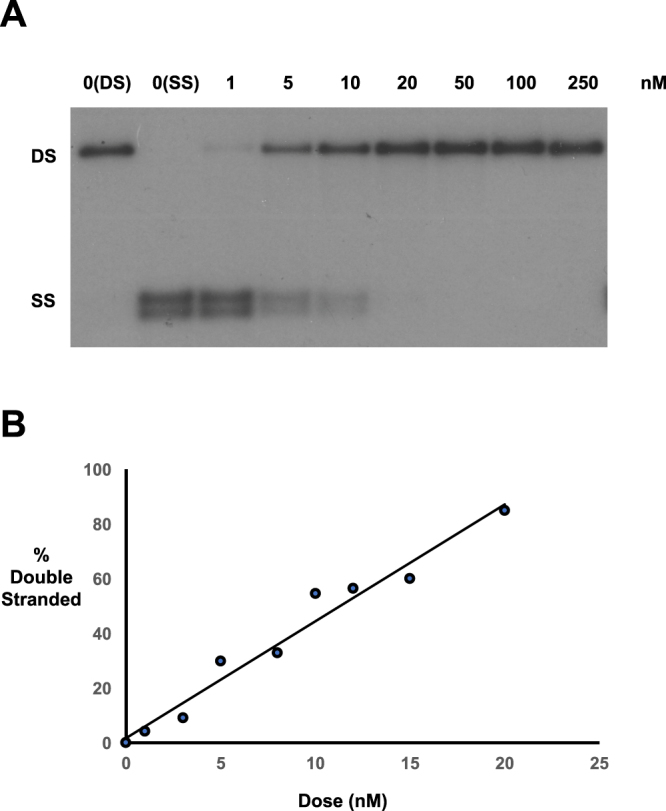
DNA interstrand cross-linking by SG3199 in naked DNA. (**A**) Representative autoradiograph showing double stranded (DS) and single stranded (SS) DNA. 0(DS) is non-denatured, untreated DNA. All other samples treated with the indicated dose of SG3199 are denatured prior to loading on the neutral agarose gel. (**B**) Quantitation of the % double stranded (cross-linked) DNA following SG3199 treatment.

PBD dimers were designed as efficient DNA interstrand cross-linking agents. The ability of SG3199 to form covalent DNA interstrand cross-links in naked linear plasmid DNA (approximately 4000 base pairs) was determined using an agarose gel-based assay^24^ (Fig. 4). A representative gel autoradiograph is shown in Fig. 4A. Following complete denaturation of double stranded DNA to single stranded, the presence of dose-dependent DNA interstrand cross-links, which prevent the complete separation of the two DNA strands and allow re-naturation to double stranded DNA in the neutral agarose gel, are observed. Using a more restricted dose range, densitometric quantitation of the percent double stranded DNA clearly shows a linear increase in cross-linking over the range 1 to 20 nM (Fig. 4B).

### DNA interstrand cross-link formation by SG3199 in cells

**Figure 5:**
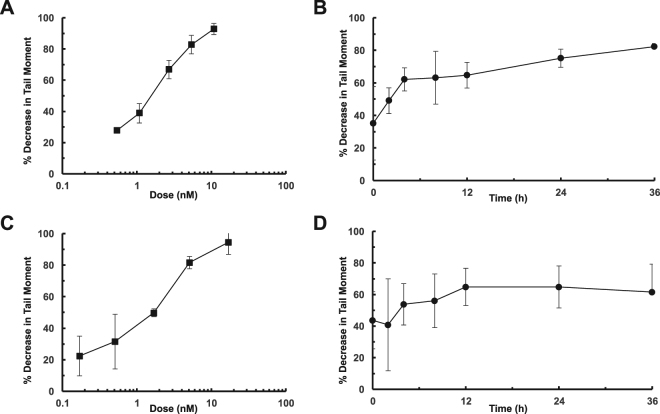
DNA interstrand cross-linking in cells by SG3199. (**A**) Dose response of cross-linking following a 2 h treatment of LNCaP cells with SG3199 followed by 24 h post-incubation in drug-free medium. (**B**) Time course of cross-linking following a 2 h SG3199 treatment in LNCaP cells at 2.5 nM. (**C**) Dose response of cross-linking following a 2 h treatment of NCI-N87 cells with SG3199 followed by 24 h post-incubation in drug-free medium. (**D**) Time course of cross-linking following a 2 h SG3199 treatment in NCI-N87 cells at 1.7 nM.

DNA interstrand cross-linking by SG3199 was measured in cells using a modification of the single cell gel electrophoresis (comet) assay^25^. Cross-linking was expressed as the percentage decrease in the Olive Tail Moment (OTM) compared to control irradiated cells. Following a 2-hour exposure of human prostate LNCaP cells to SG3199, followed by a 24 hour post-incubation in drug-free medium, a dose-dependent increase in DNA interstrand cross-linking was observed (Fig. 5A). The time course of cross-link formation was established following 2.5 nM SG3199 for two hours (Fig. 5B). The majority of cross-links had already formed during this incubation period and they continued to increase up to four hours post-incubation. The level of cross-linking then persisted over 36 hours. A similar pattern of cross-link formation and persistence was observed in human gastric cancer cell line NCI-N87 (Fig. 5C,D).

### Pharmacology of SG3199

The *in vitro* binding of [^3^H]-SG3199 to the plasma proteins of rat (Sprague Dawley), cynomolgus monkey and human at concentrations of 0.8, 5 and 50 ng/mL was determined. Plasma protein binding was high in all species; rat ∼97%, cynomolgus monkey ∼90% and human ∼95%.

The potential of SG3199 to inhibit human cytochrome P450 isozymes was investigated using human liver microsomes. SG3199 at clinically relevant concentrations (<0.5 µg/mL) was considered not to be a potent inhibitor of cytochrome P450 with co-incubation and pre-incubation in the presence of NADPH. Using intact human hepatocytes, SG3199 was found to be poorly metabolised, with around 20% loss of parent after 2 hrs incubation, due to an active cellular process.

In a quantitative whole body autoradiography study, SG3199 distribution was rapid and widespread in both albino and pigmented rats (1 μg/kg target dose level), with the maximum levels observed at first sampling time (0.5 h post-dose for albino rats and 2 h post-dose for pigmented rats). SG3199 has no specific affinity for melanin containing tissues with little or no blood:brain barrier penetration. In a rat disposition study with [^3^H]-SG3199, the main route of excretion was via the faeces (97.5 ± 3.0%), biliary route, with minor urinary excretion (3.8 ± 0.3%). Excretion was rapid, with most radioactivity (83.5 ± 5.8%) recovered by 24 h post dose.

**Figure 6:**
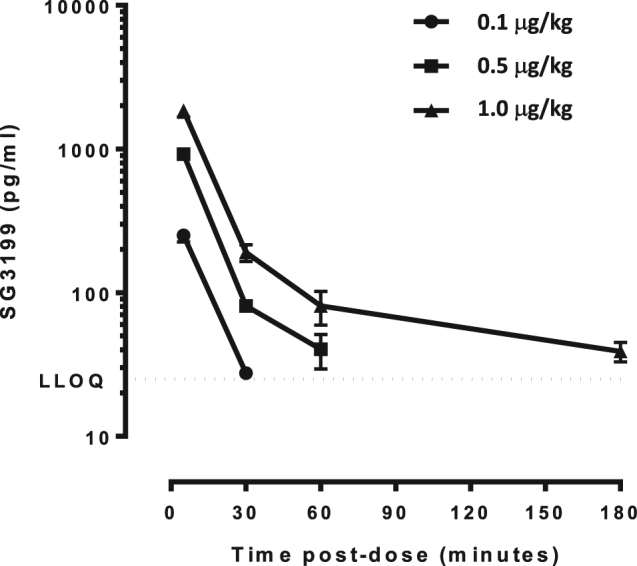
Pharmacokinetics of SG3199 in the rat following a single administration at the doses indicated. LLOQ is the lower limit of quantification.

Following i.v. administration at 0.1, 0.5 and 1 μg/kg, SG3199 showed a very rapid clearance in rats (Fig. 6). Systemic exposure to SG3199 increased in a generally dose-proportional manner, in males and females, with no evidence of accumulation over the duration of the study. Due to rapid clearance, it was not possible to calculate T_1/2_ and clearance at every dose. In the 0.5 and 1 μg/kg dose groups, the rapid clearance was between 1000 & 1500 mL/h/kg, with a T_1/2_ between 8 and 42 minutes.

## Discussion

Several important properties of the mechanism of action and pharmacology of PBD dimer SG3199 contribute to its emerging clinical success as a warhead in next-generation ADCs. Firstly, it is potently cytotoxic, showing activity across a wide range of both solid tumour and haematological cancers. No clear differential sensitivity was observed and the two least sensitive tumour cell lines still had GI_50_ values of 1 nM. This is an important factor for the utility of an ADC warhead across multiple tumour pathologies and is in contrast to tubulin inhibitor-based warheads which are less effective against some tumour types^26^. The high potency of SG3199 is also an important advantage in ADCs utilising payload tesirine being able to treat tumours with low copy number antigen targets. Previously reported PBD dimers SG2000^10^ and SG2057^17^ have a C2-*exo*-methylene group. The current study demonstrated that moving the double bond inside the PBD C-ring, as in SG3199, maintains the cytotoxic potency.

In the current study, haematological cell lines were, in general, more sensitive to SG3199 than solid tumour cell lines. It is to be noted that, for practical reasons, the cytotoxicity assay readout used for adherent solid tumour cells (CellTiter-Glo®) and suspension liquid tumour cell lines (CellTiter 96® AQueous One Solution Cell Proliferation) were different. The activity of SG3199 was evaluated in two solid tumour and one haematological cell line using both methods giving comparable results between the two assay readouts, eliminating this as a possible explanation. The data are consistent with previous results with other PBD dimers, including SG2000 in the NCI 60 cell line screen, which showed exquisite potency in haematological cell lines^10^.

The ability of SG3199 to maintain activity in tumour cells expressing multidrug resistance mechanisms is another important advantage compared to other ADC warheads such as calicheamicin^27^. The increased sensitivity to SG3199 of cells defective in homologous recombination repair and DNA repair protein ERCC1, as shown previously for SG2000^23^, suggest a possible widening of therapeutic index in patients with tumours harbouring these defects, and indicates potential biomarkers of response.

The potent activity of SG3199 is believed to derive from its ability to form highly cytotoxic DNA interstrand cross-links in cells. These cross-links form rapidly in cells in contrast to those produced in the major groove of DNA by conventional DNA cross-linking agents such as cisplatin and melphalan, which reach a peak of interstrand cross-linking at 9 hours and 16 hours, respectively, following a 2-hour drug exposure^28^. Another critical factor is the persistence of SG3199-induced cross-links compared to other DNA cross-linking agents. The non-distorting nature of the cross-links in the DNA minor groove contributes to this persistence by evading excision repair mechanisms^23^, giving this ADC warhead the important ability to kill slowly proliferating cells within tumours including tumour initiating cells^29^, although this property also gives the possibility of toxicity to quiescent cells if efficient tumour targeting of the ADC in not achieved.

The importance of the DNA interstrand cross-link as a critical cytotoxic lesion is well established for many conventional chemotherapeutic agents such as the nitrogen mustard and platinum classes^30,31^. Due to the ability to measure DNA interstrand cross-links in cells at pharmacologically relevant doses, a relationship between DNA cross-link formation and *in vitro* cytotoxicity and *in vivo* antitumour activity is observed for PBD dimers such as SG2000^10,11^ and ADCs delivering PBD dimers^20,21^. In addition, DNA interstrand cross-linking correlated with systemic exposure to SG2000 in a phase I study^13^. Studies using HPLC/MS and short oligonucleotides of defined sequence have clearly indicated that PBD dimers such as SG2000 can also form sequence selective intrastrand cross-link and mono-alkylated adducts, in addition to interstrand cross-links^9^. The abundance of these adducts compared to interstrand cross-links has not been determined for SG3199, and the contribution of these adducts to the efficacy and cytotoxicity of SG3199 cannot be determined in the absence of assays to quantitate the formation and persistence of these adducts in cells at pharmacologically relevant doses.

In addition to its high potency, SG3199 has a very short half-life, an important property in the context of an ADC warhead. ADCs with the enzyme cleavable linker-containing payload tesirine, which employs SG3199, have been shown to have a significant bystander effect^20,21^. This is an important advantage when targeting tumours with a heterogenous target antigen expression. The short half-life of the released SG3199 warhead, however, ensures that the bystander effect is restricted and that systemic accumulation of free drug, which could contribute to off-target toxicity, is limited. In addition, ADCs often have a very long half-life in circulation (10–14 days in the case of Rova-T in humans^19^). The very short half-life of SG3199 ensures that any premature release in circulation would also not result in accumulation of SG3199 to levels that cause systemic toxicity.

In summary, SG3199 has several properties which contribute to its success as an ADC warhead. It is the warhead component of tesirine^18^ which is the payload of several ADCs including rovalpituzumab tesirine, ADCT-301, ADCT-402, ADCT-401/MEDI3726 and ADCT-502 currently in different stages of clinical development.

## Methods

### Synthesis of SG3199

Following synthesis as outlined in Fig. 1B, chromatography (DCM/MeOH gradient) was required to remove all traces of Pd and acids, to prevent undesired impurity formation (such as aromatisation of the pyrrole rings) upon storage. The same synthesis was used to synthesise the deuterated D-10 analogue of SG3199 used in PK studies. SG3199 stock solutions at 10 mM in DMSO were stored at −20 °C in glass vials.

### Human cell lines

The source of the cell lines used in this study and the growth conditions for each cell line are shown in Supplementary Table S1.

### Cytotoxicity assays

Adherent cells grown to 80–90% confluence in T75 flasks were washed with PBS (10 ml) then incubated with Trypsin/EDTA (3 ml) at 37 °C in the CO_2_-gassed incubator for up to 15 min until detached. The cell suspension was diluted to 10 ml with growth medium and centrifuged at 600 g for 3 min. The cell pellet was re-suspended in 10 ml growth medium by repeated pipetting. To count the cell number and viability, trypan blue (1 vol) was added to cell suspension, mixed and measured by using an automated cell counter (LUNAII™, Logos Biosystems, Inc.). Cell counting was performed in duplicate.

Cells (1 × 10^5^/ml in growth medium) were dispensed (2 ml) in 15 ml tubes and SG3199 dilutions or DMSO (10 µl) were added and mixed. Each suspension was dispensed (100 µl/well) into 4 replicate wells of a white 96-well microplate and incubated in a 37 °C CO_2_-gassed incubator. Each assay was carried out in triplicate.

At the end of the incubation period (three cell doubling times, see Supplementary Table S1 for individual cell line incubation times), cell viability was measured by CellTiter-Glo® assay. Microplates were removed from the 37 °C incubator for 30 min to cool to room temperature. CellTiter-Glo® (Promega G7571) was dispensed (100 µl/well) into each well, the microplate agitated 2 min on an orbital shaker, left for 10 mins at room temperature to stabilise the luminescent output, then read on an EnVision® Multi-label Plate Reader (Perkin Elmer) using the luminescence protocol.

For suspension cultures, cells (1 × 10^5^cells/ml in growth medium) were dispensed (0.5 mL per tube), and to each was added a warhead dilution in DMSO (2.5 µl, as above). Each tube was gently vortexed, and then cells dispensed (100 µl per well, 4 wells per dilution) into a 96-well flat bottom microwell plate (Fisher #TKT-180-070U). The cells were incubated at 37 °C, 5% CO_2_, for 5 days, before adding 20 µl MTS solution (CellTiter 96® AQueous One Solution Cell Proliferation Assay: Promega #G3581), incubation for 4 hours at 37 °C, and measuring the well optical densities at 490 nM.

Cell survival (%) was calculated from the mean well luminescence or optical density reading in the four SG3199-treated wells, compared to the mean reading in the four control wells receiving culture medium plus DMSO only (100%). Dose-response curves were generated from the mean data of three replicate experiments. The IC_50_ was determined by fitting each of the three replicate data sets to “sigmoidal dose-response curves with variable slope” using GraphPad Prism software (GraphPad, San Diego, CA). The three IC_50_ values were then averaged and the standard error of the mean determined.

### Agarose gel-based determination of DNA interstrand cross-linking in naked DNA

This method is as previously described^24^. Briefly, pBR322 plasmid DNA was linearised by digestion with *Hind III* and dephosphorylated by treatment with bacterial alkaline phosphatase. The DNA was 5′-end-labelled using T4 polynucleotide kinase and [gamma-32P] ATP (6000 Ci/mmol 10 mCi/ml EasyTide, 9.25MBq).

Following precipitation and removal of unincorporated ATP, the DNA was resuspended in sterile double-distilled water at 125 ng/µL and approximately 10 ng of labelled DNA was used for each experimental point. Drug reactions were performed in 25 mM triethanolamine, 1 mM EDTA (pH 7.2) at 37 °C for 1 hr. Reactions were terminated by the addition of an equal volume of stop solution (0.6 M sodium acetate, 20 mM EDTA, 100 μg/mL tRNA) and the DNA was precipitated by the addition of 3 vols 95% ethanol. Following centrifugation and removal of supernatant the DNA pellet was dried by lyophilisation.

Samples were dissolved in 10 µL of freshly made alkaline strand-separation buffer (1% Sodium Hydroxide, 6% Sucrose, 0.04% bromophenol blue), and vortexed for 3 minutes and immediately loaded onto the gel. Control non-denatured samples were dissolved in 10 µL 6% sucrose, 0.04% bromophenol blue and loaded. Electrophoresis was performed on 20 cm long 0.8% agarose submerged horizontal agarose gel at 40 V for 16 h. The gel and running buffer was 40 mM Tris, 20 mM acetic acid and 2 mM EDTA (pH 8.1). Gels were dried at 80 °C onto double layer Whatman 3MM on a Hoefer GD 2000 gel drier connected to a vacuum. Autoradiography was performed with double-sided X-Ray film which was scanned and the percentage double-stranded (cross-linked) DNA calculated using the ImageJ software package.

### DNA interstrand cross-link determination in cells using the single cell gel electrophoresis (comet) assay

Cell lines were incubated with SG3199 for 2 h at 37 °C, washed, re-suspended in medium and incubated at 37 °C over a further time course (0–36 h). After each time point, cells were centrifuged, re-suspended in freezing medium (FBS with 10% DMSO) and frozen at −80 °C. To determine percentage cross-linking of increasing doses of SG3199 on expressing cell lines each ADC dilution was added to cells for two hours at 37 °C. Cells were washed, re-suspended in culture medium, dispensed into 24 well plates, incubated for 24 h at 37 °C and harvested, as above. Frozen cells were thawed on ice, re-suspended in cold RPMI (Sigma) and counted. Cell suspensions (2–3 × 10^4^ cells/ml), excepting un-irradiated controls, were irradiated (15 Gy) and kept on ice. The Comet assay was performed as previously described^25^. Slides were reviewed under a 20x objective on an epi-fluorescence microscope equipped with: Hg arc lamp; 580 nm dichroic mirror; and 535 nm excitation and 645 nm emission filters suitable for visualising propidium iodide staining with a minimum of 50 Comet images acquired per treatment condition. The Olive Tail Moment (OTM^32^) was determined as the product of the tail length and the fraction of total DNA in the tail as recorded by Komet 6 software (Andor Technology, Belfast, UK) and the percentage reduction in OTM calculated according to the formula:

$$ \% \,{\rm{decrease}}\,{\rm{in}}\,{\rm{tail}}\,{\rm{moment}}=[1-({\rm{TMdi}}-{\rm{TMcu}})/({\rm{TMci}}\,\mbox{--}\,{\rm{TMcu}})]\ast 100$$

TMdi = Tail Moment drug irradiated; TMcu = TM control un-irradiated; TMci = TM control irradiated.

Analysis was performed on Microsoft Excel and GraphPad.

### Plasma protein binding

The *in vitro* binding of [^3^H]-SG3199 to the plasma proteins of rat (Sprague Dawley), cynomolgus monkey and human at the concentrations of 0.8, 5 and 50 ng/mL was determined by equilibrium dialysis.

### Metabolism of SG3199 in human hepatocytes

Cryopreserved human hepatocytes (0.5 × 10^6^ viable cells/mL, lysed and intact) were incubated with at SG3199 (3 μM) or control at 37 °C (final DMSO concentration in the incubation was 0.25%). Samples (50 μL) were obtained from the incubation mixture at 0, 10, 20, 40, 60 and 120 min and added to methanol, containing internal standard, (100 μL) to stop the reaction. The samples were centrifuged (2500 rpm at 4 °C for 30 min) and the supernatants at each time point pooled for analysis by LC-MS/MS.

### Cytochrome P450 inhibition

The effect of increasing concentrations of SG3199 on the metabolism of known probe substrates for each of the major human drug metabolising cytochrome P450 was investigated using human liver microsomes. For each isoform the following substrates were used to assess enzyme activities: CYP1A2 (phenacetin), CYP2A6 (coumarin), CYP2B6 (bupropion), CYP2C8 (paclitaxel), CYP2C9 (tolbutamide), CYP2C19 (*S-*mephenytoin), CYP2D6 (bufuralol), CYP2E1 (chlorzoxazone) and CYP3A4 (testosterone and midazolam).

### Tissue distribution and disposition

Animal procedures (including experimental protocol approval) were completed according to the Charles River Laboratories Edinburgh standard operating procedures. Experimental protocols were approved by the Charles River Laboratories Edinburgh animal ethics committee. Charles River Laboratories Edinburgh is regulated to perform scientific procedures under specific licences issued by the UK Home Office according to the Animals (Scientific Procedures) Act 1986 (compliant with associated EU and US guidelines).

The tissue distribution of total radioactivity following i.v. administration of [^3^H]-SG3199 to male Sprague Dawley (albino) and Lister Hooded (pigmented) rats at a target dose level of 1 µg/kg was determined by quantitative whole body autoradiography. The excretion and plasma kinetics of total radioactivity following i.v. administration of [^3^H]-SG3199 was determined in Sprague Dawley rats. Male rats received a single intravenous administration of [^3^H]-SG3199, with sampling of urine, faeces, expired air and blood, where appropriate, at pre-determined time points up to 168 h post dose.

### Assessment of SG3199 pharmacokinetics

The determination of free SG3199 in rat serum was performed by LC-MS/MS and uses as internal standard (IS) SG3199-d10, deuterium labelled SG3199, using a developed and GLP-compliant validated assay.

Isolation of SG3199 (and spiked SG3199-d10) from rat serum was performed by reduction with cyanoborohydride overnight and followed by off-line solid phase extraction using oasis HLB 96-well µElution Plate, 30 µm Particle Size. The purified samples were analyzed by LC-MS/MS using an AB Sciex Qtrap 5500 LC-MS/MS system with a Zorbax SB-AQ Rapid Resolution HT (2.1 × 50 mm; 1.8 µm) column. Mobile phase A was 10 mM ammonium formate pH4.0:Acetonitrile (90:10% v/v) and Mobile phase B was 10 mM ammonium formate pH4.0:Acetonitrile (10:90% v/v).

As part of a series of serum stability studies it was noted that SG3199 binds slowly and irreversibly to a component of serum (could not be disrupted by the current LC-MS/MS sample pretreatment procedure). As such any LC-MS/MS measure of SG3199 is considered to be free SG3199.

## Electronic supplementary material

Supplementary Information
