# Supplementary material for: Pre-clinical pharmacology and mechanism of action of SG3199, the pyrrolobenzodiazepine (PBD) dimer warhead component of antibody-drug conjugate (ADC) payload tesirine
Source: Sci Rep. 2018 Jul 11;8:10479. doi: 10.1038/s41598-018-28533-4 (PMC6041317; doi:10.1038/s41598-018-28533-4)
Supplement: Supplementary file 1 — Supplementary Information [file 41598_2018_28533_MOESM1_ESM.pdf]

SUPPLEMENTARY INFORMATION

Pre-clinical pharmacology and mechanism of action of SG3199, the pyrrolobenzodiazepine (PBD) dimer warhead component of antibody-drug conjugate (ADC) payload tesirine

John A Hartley[1,2,*], Michael J Flynn[1], John P Bingham[1], Simon Corbett[1,2], Halla Reinert[1], Arnaud Tiberghien[2], Luke A Masterson[2], Dyeison Antonow[2], Lauren Adams[2], Sajidah Chowdhury[2], David G Williams[2], Shenlan Mao[3], Jay Harper[3], Carin EG Havenith[4], Francesca Zammarchi[4], Simon Chivers[4], Patrick H van Berkel[4] and Philip W Howard[2].

Supplementary Table S1. Source of cell lines, cell growth media and SG3199 incubation times for cytotoxicity determinations.

Cell Line	Source: catalogue number	Cell Growth Medium		SG3199 Incubation Time (Days)
		Basal Medium	Serum % v/v	
Calu-3	ATCC: HTB-55	Minimal Essential Medium Eagle. Sigma #M4655	10% HyClone GE Healthcare #SH30070)	6
Capan-1	ATCC: HTB-79	IMDM Thermo #12440053	20% HyClone	8
CFPAC-1	ATCC: CRL-1918	IMDM	10% HyClone	6
JIMT-1	DSMZ: ACC 589	DMEM Thermo #61965-026	10% HyClone	5
MDA-MB-468	ATCC: HTB-132	RPMI 1640. Thermo #61870-044	10% HyClone	3
NCI-H520	ATCC: HTB-182	RPMI 1640.	10% HyClone	4
NCI-H522	ATCC: CRL-5810	RPMI 1640.	10% HyClone	6
NCI-N87	ATCC: CRL-5822	RPMI 1640.	10% HyClone	6
RT-112	DSMZ: ACC 418	RPMI 1640.	10% HyClone	4
SK-BR-3	ATCC: HTB-30	McCoy's 5A. Thermo #36600-021	10% HyClone	6
SK-OV-3	ATCC: HTB-77	McCoy's 5A.	10% HyClone	5
T-47D	ATCC: HTB-133	RPMI 1640.	10% HyClone	4
TOV21G	ATCC: CRL-11730	RPMI 1640.	10% HyClone	4
LNCaP	ATCC® CRL-1740™	RPMI 1640 (Thermo Fisher 61870-010)	10% HyClone	6

LNCaP C4-2	Gift from Dr Neil Bander	RPMI 1640	10% HyClone	6
CWR22Rv1	ATCC® CRL-2505™	RPMI 1640	10% HyClone	5
MDA PCa 2b	ATCC® CRL-2422™	BRFF1 media (USBio p9054) + GlutaMax I (Gibco 35050-061). Grow on Poly-D-lysine (Sigma P1149)-coated plates	20% HyClone	6
PC3	ATCC® CRL-1435™	RPMI 1640	10% HyClone	6
P4E6	SIGMA 10112301	StemLine® Keratinocyte Medium II (Sigma S0196); + Keratinocyte Growth Supplement (Sigma S9945);+ GlutaMax I	10% HyClone	7
DU145	ATCC® HTB-81™	Minimum Essential Medium Eagle (Sigma M4655)	10% HyClone	5
Daudi	ATCC<br>CRL-1942	RPMI 1640	10% FBS (Life Technologies 26140-079)	5
Ramos (RA 1)	ATCC<br>CRL-1596	RPMI 1640	10% FBS	5
DOHH-2	DSMZ<br>ACC47	RPMI 1640	10% FBS	5
Granta 519	DSMZ<br>ACC342	DMEM (Life Technologies 61965-026)	10% FBS	5
Namalwa	DSMZ<br>ACC24	RPMI 1640	10% FBS	5
SU-DHL-4	DSMZ<br>ACC495	RPMI 1640	10% FBS	5
Mec2	DSMZ<br>ACC500	IMDM (Life Technologies 31980-030)	10% FBS	5
Nalm-6	DSMZ	RPMI 1640	10% FBS	5

	ACC128			
SUP-T1	DSMZ<br><br>ACC140	RPMI 1640	10% FBS	5
KM-H2	DSMZ<br><br>ACC8	RPMI 1640	10% FBS	5
SKOV3-TR	Gift from Dr Michael Seiden	RPMI 1640	10% FBS	3
MDA-MB-231	NIH	RPMI 1640	10% FBS	3
MDA-MB-231-MDR1	NIH	RPMI 1640	10% FBS	3
CHO-AA8	Gift from Dr M Stefanini	F12-Ham HEPES	10% FBS	3
CHO-UV96	Gift from Dr M Stefanini	F12-Ham HEPES	10% FBS	3
CHO-IRS1SF	Gift from Dr M Stefanini	F12-Ham HEPES	10% FBS	3

Legend: SG3199 incubation time is three cell doubling times. IMDM: Iscove's Minimal Dulbecco's Medium. DMEM: Dulbecco's Minimal Essential Medium.
